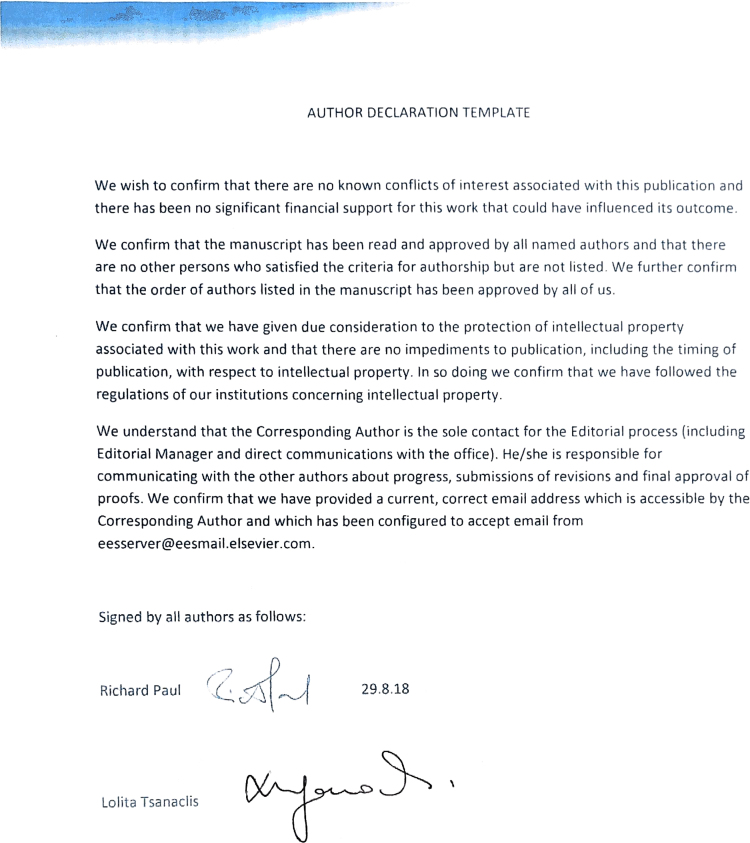# Data on ethyl glucuronide and cocaethylene concentrations in the hair of cocaine users

**DOI:** 10.1016/j.dib.2018.09.043

**Published:** 2018-09-19

**Authors:** Richard Paul, Lolita Tsanaclis

**Affiliations:** a Bournemouth University, Poole, Dorset BH12 5BB, United Kingdom; b Cansford Laboratories, 1a Pentwyn Business Centre, Wharefdale Road, Cardiff CF23 7HB, United Kingdom

**Keywords:** Ethyl glucuronide, Cocaethylene, Hair testing, Cocaine

## Abstract

We present data on ethyl glucuronide and cocaethylene concentrations from the hair of cocaine users. Head hair from 64 subjects, previously tested for cocaine, cocaethylene, benzoylecogonine and anhydroecgonine methyl ester (AEME), were subsequently analysed for Ethyl Glucuronide (EtG). Samples were prepared by solid phase extraction and analysed using gas chromatrography coupled to tandem mass spectrometry. The dataset is made available to allow analysis of possible correlation between cocaethylene and ethyl glucuronide or between other metabolites presented in the data.

**Specifications table**

**Table:** 

Subject area	*Chemistry*
More specific subject area	*Forensic toxicology*
Type of data	*Table, figure*
How data was acquired	*Head hair samples were analysed using gas chromatography coupled to tandem mass spectrometry to acquire identification and concentration of alcohol and cocaine metabolites.*
Data format	*Analysed*
Experimental factors	*Hair sample length, hair preparation and analysis methodology is consistent throughout.*
Experimental features	*Hair samples from 64 subjects were previously analysed for cocaine, cocaethylene, benzoylecogonine and anhydroecgonine methyl ester (AEME). The same hair samples were subsequently tested for ethyl glucuronide (EtG) to allow observation of possible correlation between cocaethylene and EtG.*
Data source location	*TrichoTech Ltd, Cardiff, U.K.*
Data accessibility	*Data is with this article*
Related research article	*n/a*

**Value of the data**

• Data is provided from 64 subjects showing cocaine, cocaethylene and other metabolite concentrations in head hair, alongside ethyl glucuronide concentrations from the same hair section.
• Trends and positive / negative correlation may be evaluated from the data.
• Data provides insight into the range of concentrations that may be observed in hair for the detected compounds.

## 1 Data

**Fig. 1:**
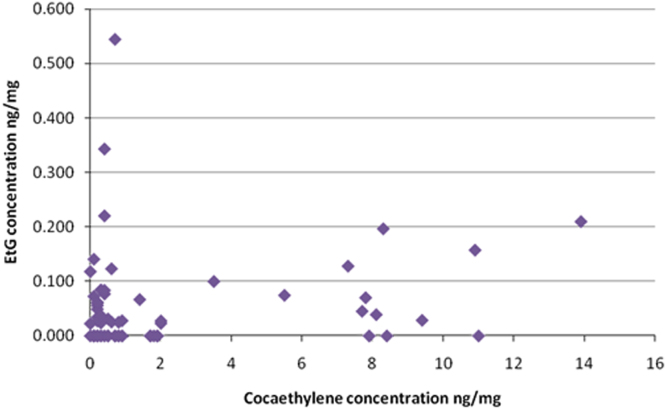
Cocaethylene concentration versus EtG concentration in 64 hair samples.

**Fig. 2:**
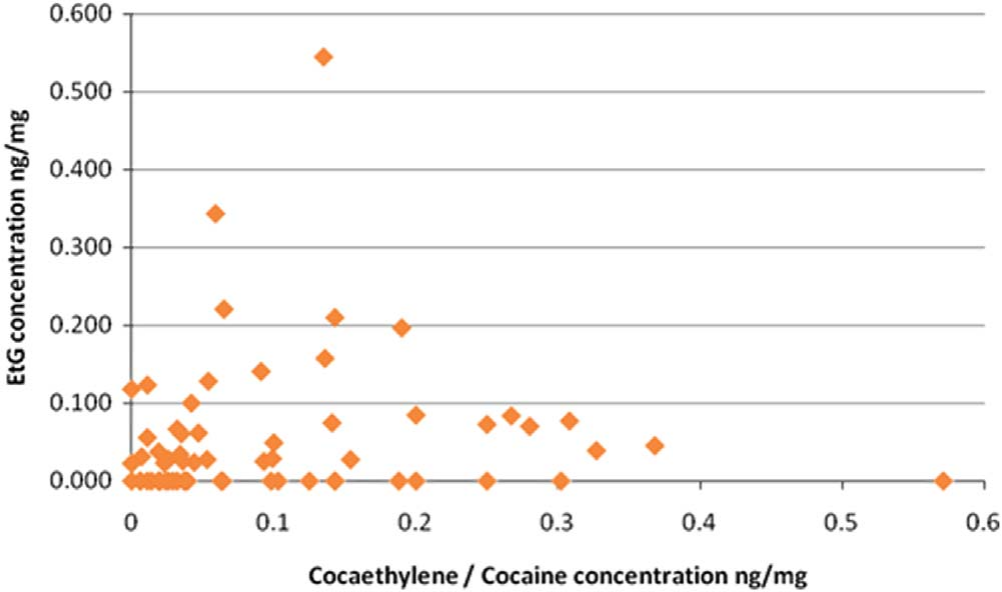
Cocaethylene/cocaine concentration versus EtG concentration in 64 hair samples.

Data were obtained from TrichoTech Ltd for hair samples from 64 subjects previously analysed for cocaine, cocaethylene, benzoylecogonine and anhydroecgonine methyl ester (AEME). The same hair samples (B samples) were subsequently tested for ethyl glucuronide (EtG) to allow observation of possible correlation between cocaethylene and EtG. A dataset of hair concentrations for these drugs and metabolites is presented. (Fig. 1, Fig. 2 and Table 1).

**Table 1:** Correlation between EtG and cocaethylene.

	Cocaethylene negative	Cocaethylene positive	Total (EtG)
EtG negative	3	25	28
EtG positive	0	36	36
Total (Cocaethylene)	3	61	64

## 2 Experimental design, materials, and methods

Hair samples from the 64 subjects were split lengthways, into two samples, creating an A and B sample. Sample A then continues through the testing procedure whilst sample B, which is identical in length and weight, remains sealed and is stored in case a sample needs to be re-analysed.

The B hair samples from 64 subjects which had been previously tested for cocaine and its metabolites were cut into 1 cm sections from the root end of the hair. These hair sections were then analysed to determine EtG concentration. By using the B sample it was possible for the data from the newly tested EtG hair samples to match the time period of their corresponding A sample which was tested for cocaine and metabolites, allowing the possibility of concentration correlation to be examined. The analysis of cocaine and metabolites was conducted at TrichoTech Ltd, a UKAS accredited facility using a SPE clean-up stage, followed by GCMS confirmation.

## 3 Preparation and analysis of hair samples

Methodology for the hair preparation, extraction and analysis is described by Paul et al. [1].

## 4 Dataset

Quantitative data from EtG, cocaine, benzoylecogonine, cocaethylene and AEME are presented. Cocaethylene / cocaine data are also presented for analysis. Hair section weights are provided. EtG concentration within the 64 subjects ranged from 0.02 ng/mg to 0.54 ng/mg with a mean concentration of 0.05 ng/mg (standard deviation 0.09 ng/mg). Cocaethylene concentrations ranged from 0.1 ng/mg to 13.9 ng/mg with a mean concentration of 2.19 ng/mg (standard deviation 3.41 ng/mg). EtG concentration plotted against cocaethylene concentration is demonstrated in Fig. 1.

Illustrating the data in this manner can lead to the incorporation of error by not separating the effect a high cocaine concentration would have on the corresponding amount of cocaethylene. For instance, if on average 17% of cocaine is converted to cocaethylene *in vivo*, then someone who consumes a very high dose of cocaine may in turn produce a larger concentration of cocaethylene. To avoid this error the ratio of cocaethylene to cocaine can be plotted against EtG concentration. A graph representing this is shown in Fig. 2.

A qualitative comparison of EtG and cocaethylene is shown in Table 1. Sixty one samples tested positive for cocaethylene whereas 36 tested positive for EtG.

## Funding

This research did not receive any specific grant from funding agencies in the public, commercial, or not-for-profit sectors.

## Figures and Tables

**Fig. S1:**